# Supplementary material for: Differences in Starvation-Induced Autophagy Response and miRNA Expression Between Rat Mammary Epithelial and Cancer Cells: Uncovering the Role of miR-218-5p
Source: Cancers (Basel). 2025 Jul 23;17(15):2446. doi: 10.3390/cancers17152446 (PMC12346175; doi:10.3390/cancers17152446)
Supplement: Supplementary file 1 [file cancers-17-02446-s001.zip › Figure S2.pdf]

Original Western Blot images for each replicate

EXPERIMENT 1

LAMP1, Beclin-1, LC3-II

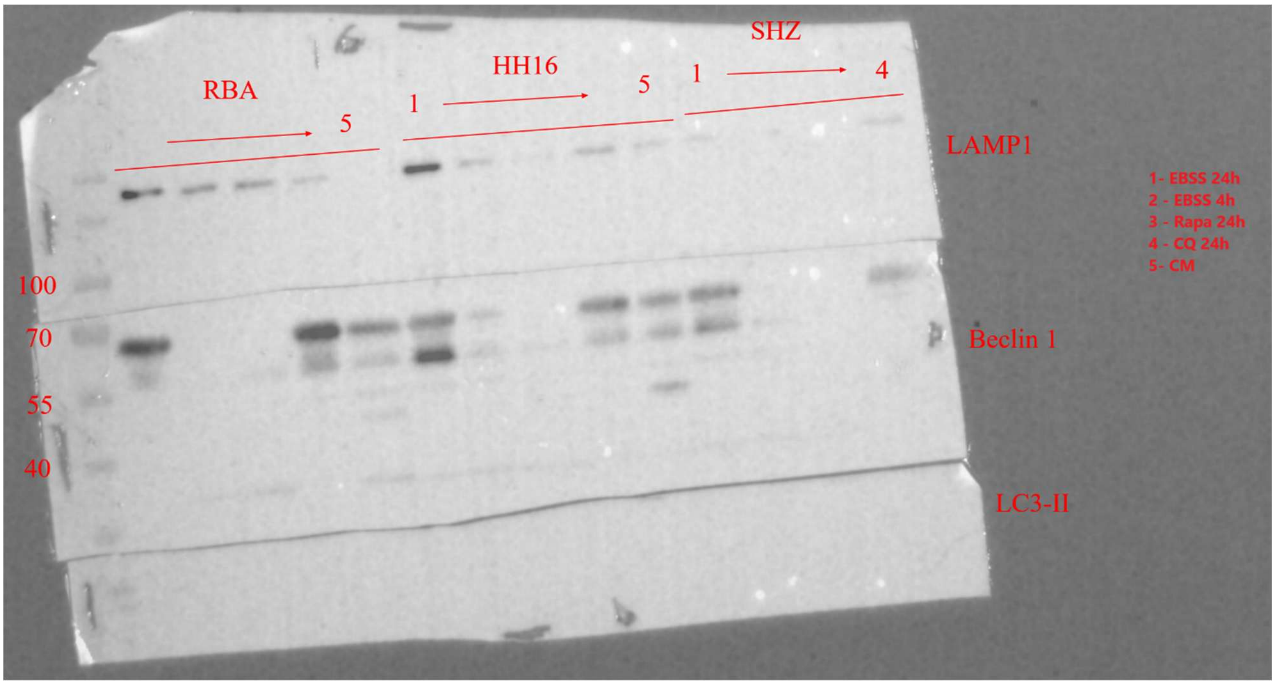

Beta-actin

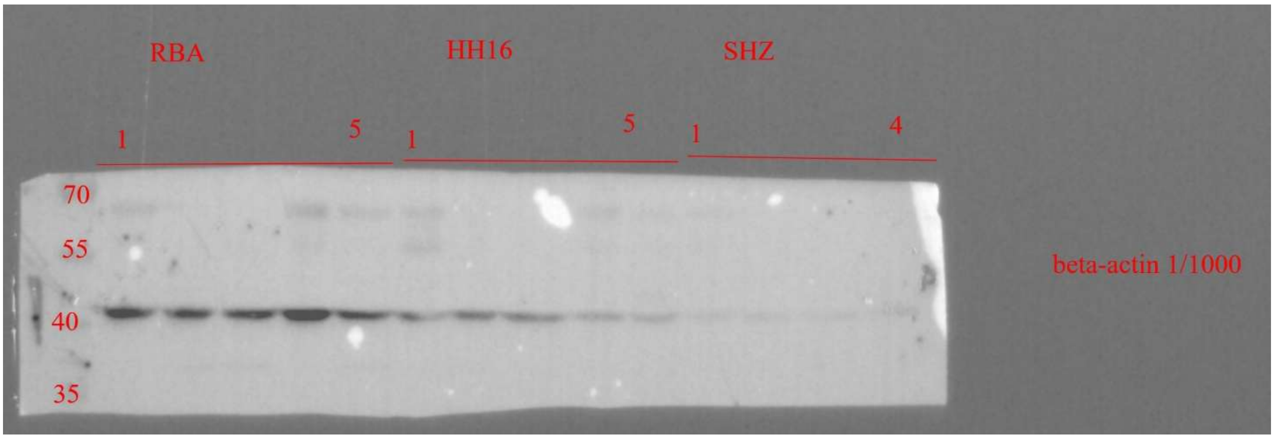

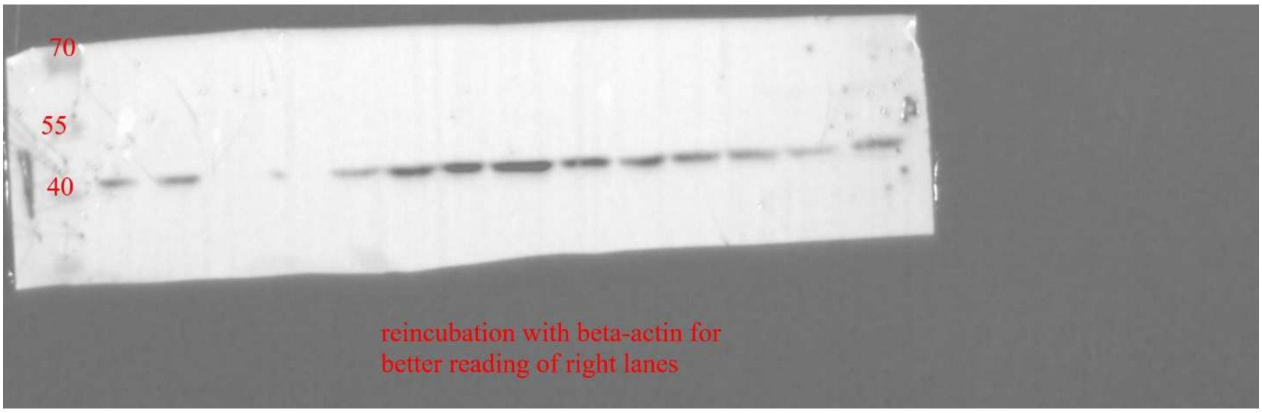

EXPERIMENT 2

Beclin-1

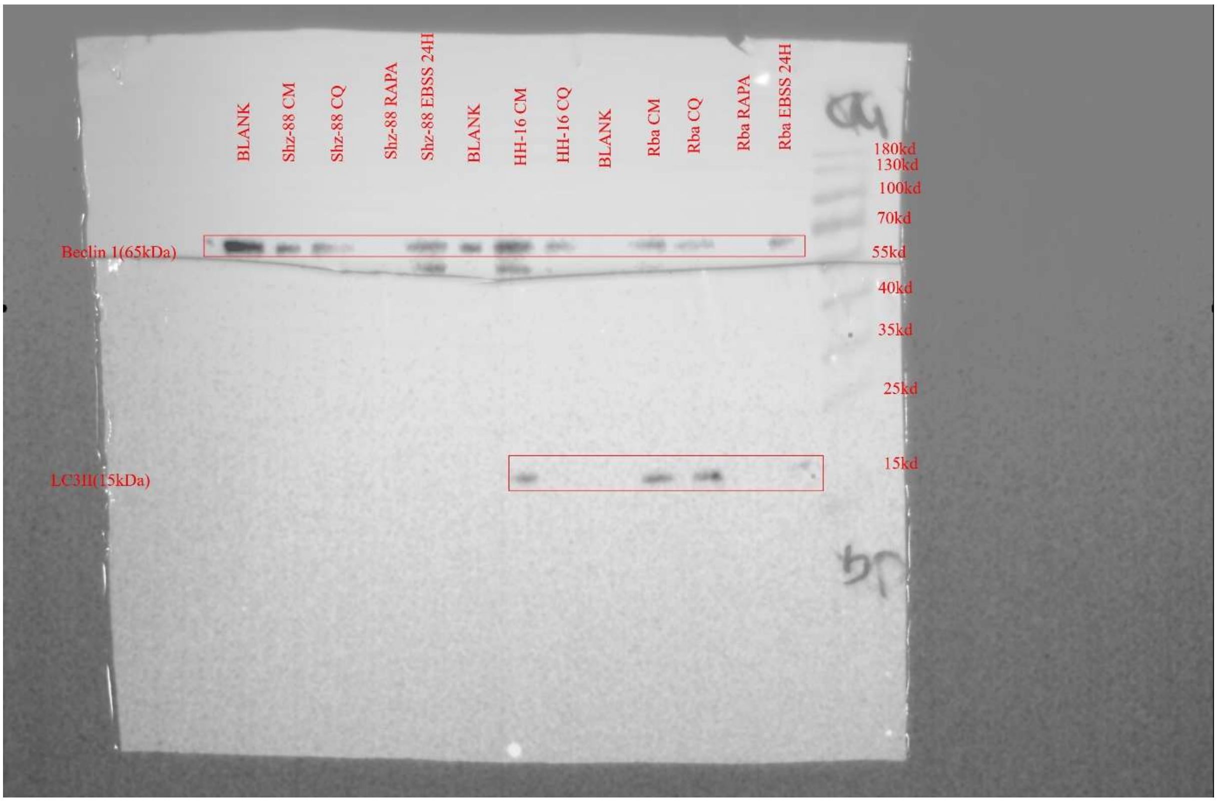

Only upper part of membrane for better quality

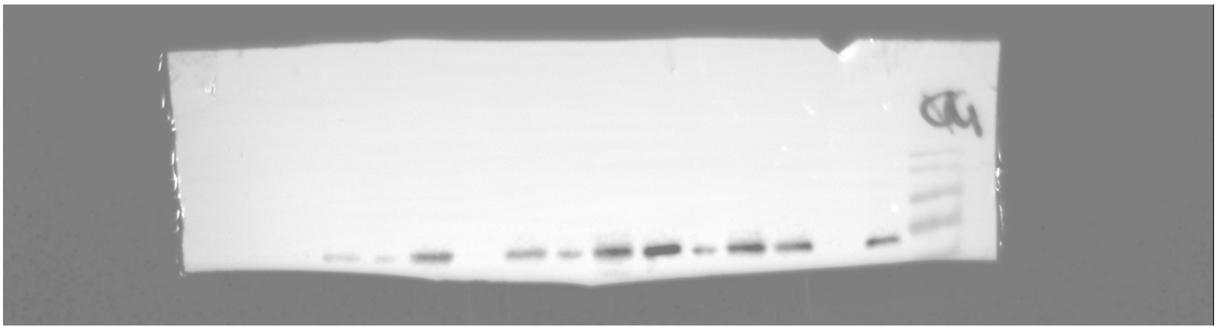

Beta-actin

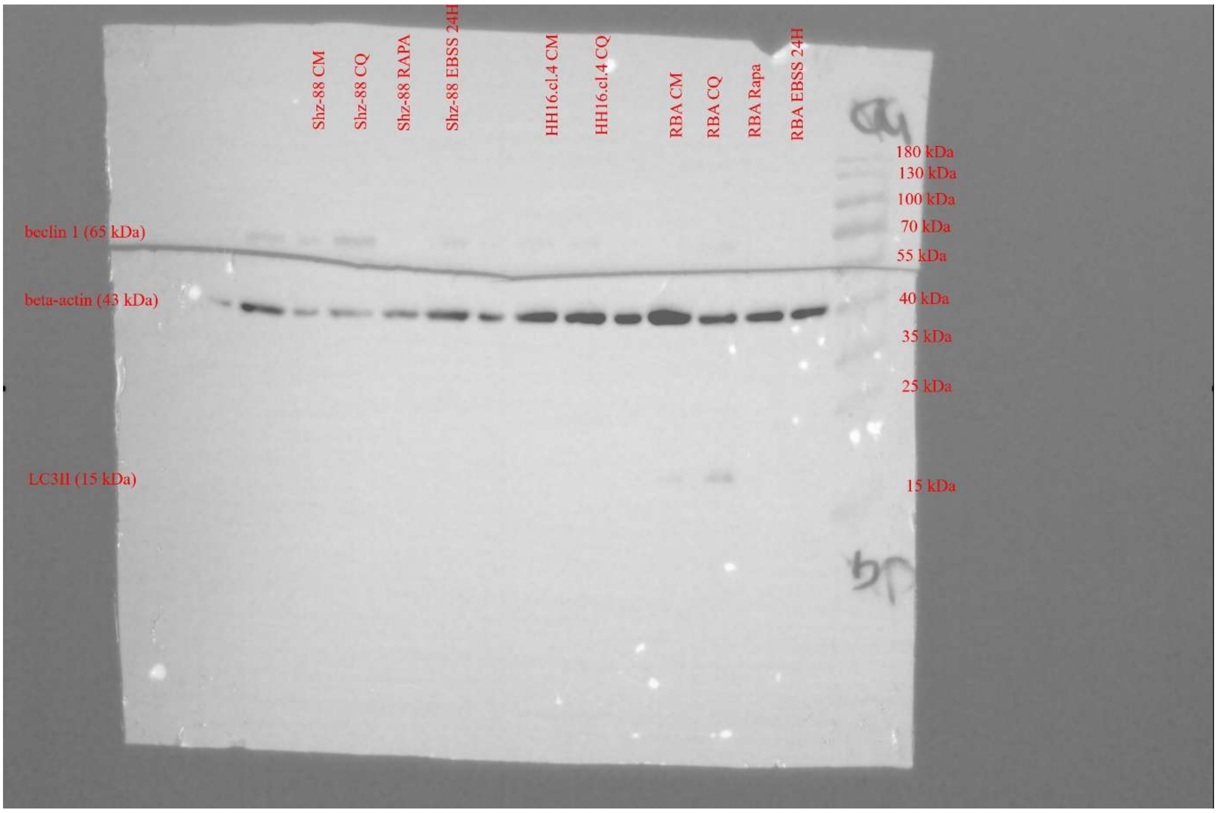
Shz-88 CM
Shz-88 CQ
Shz-88 RAPA
Shz-88 EBSS 24H
HH16.cl.4 CM
HH16.cl.4 CQ
RBA CM
RBA CQ
RBA Rapa
RBA EBSS 24H
beclin 1 (65 kDa)
beta-actin (43 kDa)
LC3II (15 kDa)
180 kDa
130 kDa
100 kDa
70 kDa
55 kDa
40 kDa
35 kDa
25 kDa
15 kDa
