# Supplementary material for: Differences in Starvation-Induced Autophagy Response and miRNA Expression Between Rat Mammary Epithelial and Cancer Cells: Uncovering the Role of miR-218-5p
Source: Cancers (Basel). 2025 Jul 23;17(15):2446. doi: 10.3390/cancers17152446 (PMC12346175; doi:10.3390/cancers17152446)

Average U6 Ct values of each sample (ca. 4ng cDNA) for each independent experiment

First run (06.04.2024)

SAMPLE	CM	EBSS	difference
RBA	13,878	13,817	0,061
HH-16	14,853	14,731	0,122
SHZ-88	16,904	16,288	0,616
MEC	15,409	16,010	-0,601

Second run (02.06.2024)

SAMPLE	CM	EBSS	difference
RBA	14,924	14,765	0,159
HH-16	15,909	15,067	0,842
SHZ-88	14,193	15,808	-1,615
MEC	15,382	16,844	-1,462

Third run (20.07.2024)

SAMPLE	CM	EBSS	difference
RBA	13,439	13,369	0,070
HH-16	13,888	14,491	-0,603
SHZ-88	13,506	13,326	0,180
MEC	15,736	16,196	-0,460

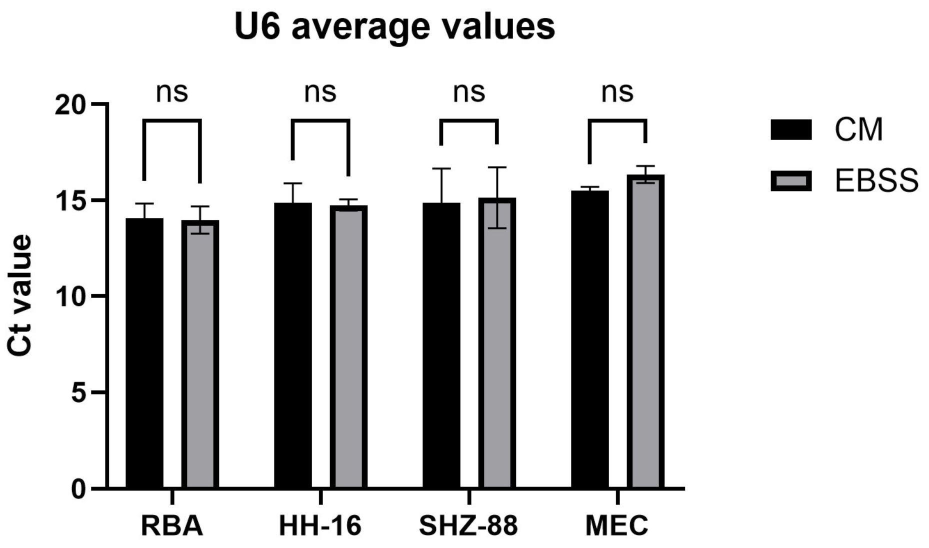

Two-way ANOVA showed that Treatment (CM vs. EBSS) was a source of significant variation (38,34%, p=0,038), however there were no significant differences in any of the cell lines in Ct value change between non-starved (CM) and starved (EBSS) groups.

Comparison of average U6, GAPDH and BETA-ACTIN Ct difference of CM- and EBSS-treated samples (29.05.2025)

SAMPLE	U6 difference	GAPDH difference	BETA-ACTIN difference
**RBA**	-0,498	0,405	0,120
**HH-16**	-0,017	-3,782	-3,025
**SHZ-88**	0,221	0,140	-2,422
**MEC**	-1,471	-2,503	-4,248
Difference between lowest and highest Ct value overall	**4,351**	**6,887**	**7,391**

Additionally, we measured the expression change in CM vs. EBSS treated cells using forward and reverse primers for two housekeeping genes (GAPDH, BETA-ACTIN) and not a single 5'-primer as expected in MiR-X-First-Strand-Synthesis Kit (mRQ3 primer).

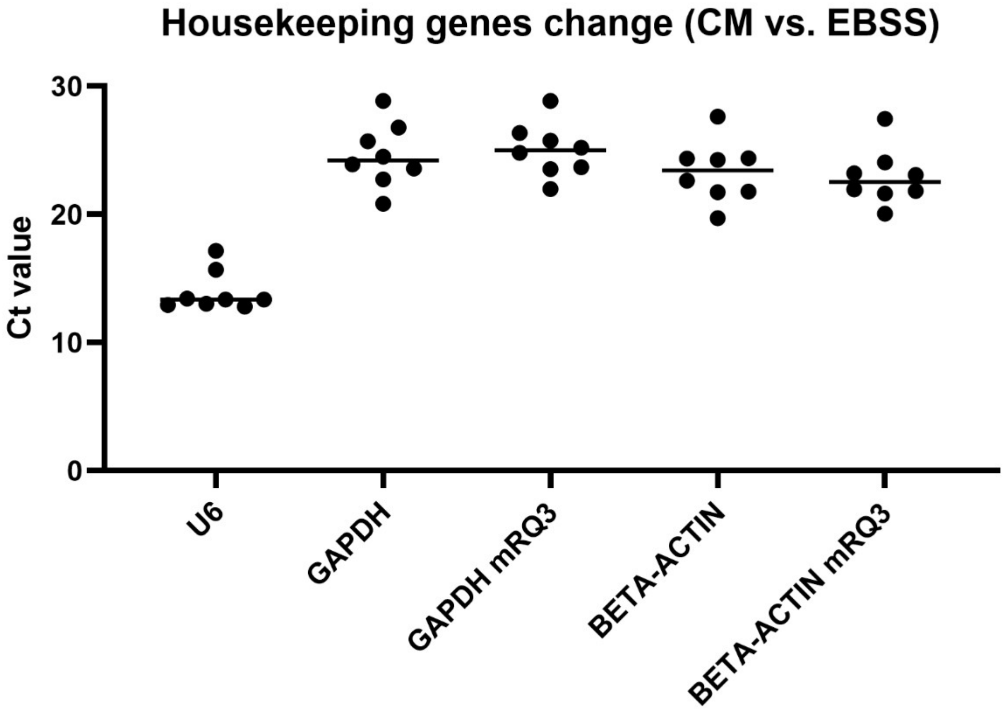

Supplement: Supplementary file 1 [file cancers-17-02446-s001.zip › Figure S3.pdf]
