# Supplementary material for: Differences in Starvation-Induced Autophagy Response and miRNA Expression Between Rat Mammary Epithelial and Cancer Cells: Uncovering the Role of miR-218-5p
Source: Cancers (Basel). 2025 Jul 23;17(15):2446. doi: 10.3390/cancers17152446 (PMC12346175; doi:10.3390/cancers17152446)
Supplement: Supplementary file 1 [file cancers-17-02446-s001.zip › Tables S1-S3.pdf]

Table S1. All primers used for the experimental miRNA expression assessment.

Selected miRNA name	Mature miRNA sequence	miRNA 5' primer
rno-miR-19b-3p	UGUGCAAAUCCAUGCAAAACUGA	TGTGCAAATCCATGCAAAACTGA
rno-miR-503-5p	UAGCAGCGGGAACAGUACUGCAG	TAGCAGCGGGAACAGTACTGCAG
rno-miR-103-3p	AGCAGCAUUGUACAGGGCUAUGA	AGCAGCATTGTACAGGGCTATGA
rno-miR-106b-5p	UAAAGUGCUGACAGUGCAGAU	TAAAGTGCTGACAGTGCAGAT
rno-miR-128-3p	UCACAGUGAACCGGUCUCUUU	TCACAGTGAACCGGTCTCTTT
rno-miR-497-5p	CAGCAGCACACUGUGGUUUGUA	CAGCAGCACACTGTGGTTTGTA
rno-miR-30d-5p	UGUAAACAUCCCCGACUGGAAG	TGTAAACATCCCCGACTGGAAG
rno-miR-9a-5p	UCUUUGGUUAUCUAGCUGUAUGA	TCTTTGGTTATCTAGCTGTATGA
rno-miR-15a-5p	UAGCAGCACAUAAUGGUUU	TAGCAGCACATAATGGTTT
rno-miR-375-3p	UUUGUUCGUUCGGCUCGCGUGA	TTTGTTCGTTCGGCTCGCGTGA
rno-miR-93-5p	CAAAGUGCUGUUCGUGCAGGUAG	CAAAGTGCTGTTCGTGCAGGTAG
rno-miR-27a-3p	UUCACAGUGGCUAAGUUCCGC	TTCACAGTGGCTAAGTTCCGC
rno-miR-195-5p	UAGCAGCACAGAAAUAUUGGC	TAGCAGCACAGAAATATTGGC
rno-miR-17-5p	CAAAGUGCUUACAGUGCAGGUAG	CAAAGTGCTTACAGTGCAGGTAG
rno-miR-98-5p	UGAGGUAGUAAGUUGUAUUGUU	TGAGGTAGTAAGTTGTATTGTT
rno-miR-15b-5p	UAGCAGCACAUCAUGGUUUACA	TAGCAGCACATCATGGTTTACA
rno-miR-20a-5p	UAAAGUGCUUAUAGUGCAGGUAG	TAAAGTGCTTATAGTGCAGGTAG
rno-miR-218a-5p	UUGUGCUUGAUCUAACCAUGU	TTGTGCTTGATCTAACCATGT
rno-miR-19a-3p	UGUGCAAAUCUAUGCAAAACUGA	TGTGCAAATCTATGCAAAACTGA
rno-miR-335	UCAAGAGCAAUAACGAAAAAUGU	TCAAGAGCAATAACGAAAAATGT
rno-miR-26b-5p	UUCAAGUAAUUCAGGAUAGGU	TTCAAGTAATTCAGGATAGGT
rno-miR-20b-5p	CAAAGUGCUCAUAGUGCAGGUAG	CAAAGTGCTCATAGTGCAGGTAG
rno-miR-30a-5p	UGUAAACAUCCUCGACUGGAAG	TGTAAACATCCTCGACTGGAAG
rno-miR-30e-5p	UGUAAACAUCCUUGACUGGAAG	TGTAAACATCCTTGACTGGAAG
rno-miR-16-5p	UAGCAGCACGUAAAUAUUGGCG	TAGCAGCACGTAAATATTGGCG
rno-miR-34a-5p	UGGCAGUGUCUUAGCUGGUUGU	TGGCAGTGTCTTAGCTGGTTGT
rno-miR-10b-5p	CCCUGUAGAACCGAAUUUGUGU	CCCTGTAGAACCGAATTTGTGT

Table S2. Housekeeping genes 5' primers used for validation of U6.

Selected gene name	5' primer
GAPDH_F	TGATTCTACCCACGGCAAGTT
GAPDH_R	TGATGGGTTTCCCATTGATGA
BETA-ACTIN_F	GGGAAATCGTGCGTGACATT
BETA-ACTIN_R	GCGGCAGTGGCCATCTC

Table S3. 5′ primers of genes used for miR-218-5p target verification.

**Selected gene name**	**5' primer**
GAPDH_F	TGATTCTACCCACGGCAAGTT
SNX18	CCAAGCAGACCAAGTTCAAG
HMGB1	GAGATCCTAAGAAGCCGAGA
Eif2ak3	CGGATACGGCATTTGGCTTG
Tp53inp2	GCTAAAGTGTTGCAACGGCA
CDKN1B	AGATACGAGTGGCAGGAGGT
RICTOR	TCCGAATACGAGGGCGGAA
BIRC5	GGATGACAACCCTATAGAGGAGC
