# Supplementary figures and images for: Differences in Starvation-Induced Autophagy Response and miRNA Expression Between Rat Mammary Epithelial and Cancer Cells: Uncovering the Role of miR-218-5p
Source: Cancers (Basel). 2025 Jul 23;17(15):2446. doi: 10.3390/cancers17152446 (PMC12346175; doi:10.3390/cancers17152446)

1st experiment - 28.02.2024

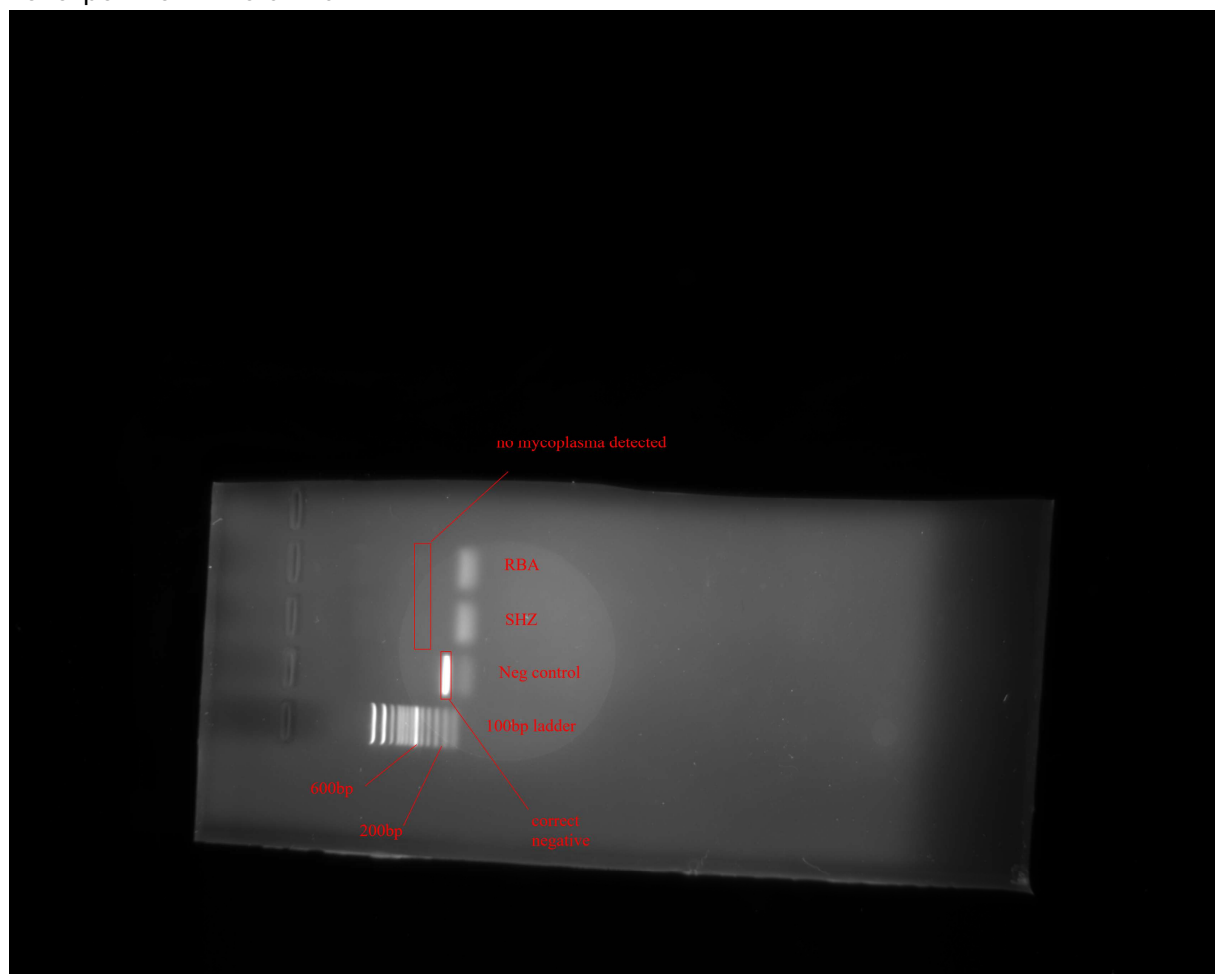

## 2nd experiment - 11.08.2024

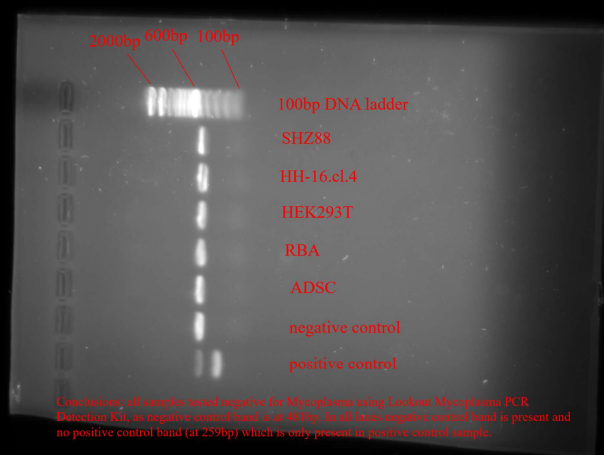

### 3rd experiment – 03.03.2025

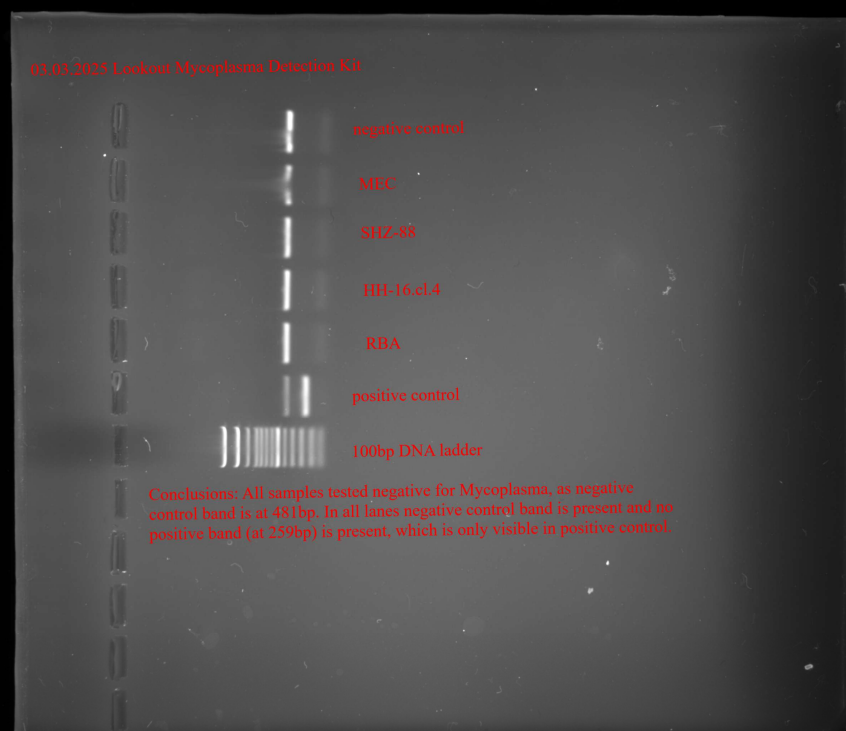

Supplement: Supplementary file 1 [file cancers-17-02446-s001.zip › Figure S1.pdf]
